# Health Disparities Experienced by Hispanic Americans with Multiple Myeloma: A Systematic Review

**DOI:** 10.1007/s44228-022-00026-2

**Published:** 2022-12-31

**Authors:** Andrea Anampa-Guzmán, Sara Taveras Alam, Inas Abuali, Samer Al Hadidi

**Affiliations:** 1 San Fernando School of Medicine, Faculty of Medicine, Universidad Nacional Mayor de San Marcos, Lima, Peru; 2 Lymphoma Section, Department of Medicine, Roswell Park Comprehensive Cancer Center, Buffalo, NY USA; 3 Hematology and Oncology, Department of Medicine, Baylor College of Medicine, Houston, TX USA; 4 Hematology and Oncology, Department of Medicine, Michael E. DeBakey Veterans Affairs Medical Center, Houston, TX USA; 5 Department of Oncology, Massachusetts General Hospital, Harvard Medical School, Boston, MA USA; 6 Myeloma Center, Winthrop P. Rockefeller Cancer Institute, University of Arkansas for Medical Sciences, Little Rock, AR USA

**Keywords:** Hispanics, Hispanic Americans, Multiple myeloma, Disparities, Health disparities, Cancer health disparities

## Abstract

Health disparities in multiple myeloma (MM) disproportionately affect minorities. Characterization of health disparities encountered by Hispanic Americans with MM is necessary to identify gaps and inform future strategies to eliminate them. We performed a systematic review of publications that described health disparities relevant to Hispanic Americans with MM through December 2021. We included all original studies which compared incidence, treatment, and/or outcomes of Hispanic Americans with other ethnic groups. Eight hundred and sixty-eight articles were identified of which 22 original study articles were included in our systematic review. The number of publications varied over time with the highest number of studies (32%) published in 2021. Most of the published studies (59%) reported worse outcomes for Hispanic Americans with MM compared to other ethnic groups. There is growing evidence that Hispanic Americans with MM are facing a multitude of disparities that require immediate attention and solutions.

## Introduction

Multiple myeloma (MM) is characterized by neoplastic proliferation of plasma cells producing a monoclonal immunoglobulin that may result in end-organ damage. MM occurs in all races and all geographic locations. The incidence varies by ethnicity; the incidence in Black populations is two to three times that of non-Hispanic Whites in studies from the United States and United Kingdom [1].

The United States Census Bureau uses Hispanic to refer to a person of Cuban, Mexican, Puerto Rican, South or Central American, or other Spanish culture or origin regardless of race and states that Hispanics or Latinos can be of any race, any ancestry, or any ethnicity [2, 3]. Hispanics are the largest and fastest-growing minority group in the U.S., and they currently make up approximately 15% of the U.S. population [4]. Hispanic Americans continue to face multiple health disparities related to MM. MM-related in-hospital mortality was significantly higher in Hispanic Americans when compared to non-Hispanic Whites and non-Hispanic Blacks. Disparities in MM care for Hispanics in the U.S. continue to persist despite recent advancements in MM therapy [5]. This can be related to limited access to care and lower utilization of effective MM therapies. There are limited data on health disparities experienced by Hispanic Americans. We aimed to review the literature for studies which reported on health disparities in Hispanic American patients with MM.

## Methods

### Literature Search and Selection

We searched EMBASE, MEDLINE/PubMed, CINAHL, Scopus, and Web of Science for English-language literature through December 2021 using the terms “multiple myeloma”, “plasmacytoma”, “Hispanic Americans”, “Hispanic”, “Spanish Americans”, “Latino”, “Latina”, “Spanish speaking”, “health disparities”, “healthcare disparities”, “disparities”. All searches were conducted during December 2021. We used methodology from the Preferred Reporting Items for Systematic Reviews and Meta-Analyses (PRISMA) Statement to guide our search, analyses, and reporting [6]. We used MESH (Medical Subject Headings) terms as they are universally used in indexing journal articles and books in the life sciences. The combinations of the terms used are listed in Table 1.

**Table 1:** Search terms

Search engine	Search terms
EMBASE	(Multiple Myeloma OR Plasmacytoma) AND (Hispanic Americans OR Hispanic OR Hispanics OR Spanish Americans OR Latino* OR Latina* OR Spanish speaking OR Healthcare Disparities)
CINAHL	(Multiple Myeloma OR Plasmacytoma) AND (Hispanic Americans OR Hispanic OR Hispanics OR Spanish Americans OR Latino* OR Latina* OR Spanish speaking OR Healthcare Disparities)
PUBMED	(“Multiple Myeloma”[MeSH] OR “Plasmacytoma”[MeSH] OR “multiple myeloma”[All Fields] OR “plasmacytoma”[All Fields]) AND (“Hispanic Americans”[ Mesh] OR “Hispanic Americans”[All Fields] OR “Hispanic”[All Fields] OR “Hispanics”[All Fields] OR “Spanish Americans”[All Fields] OR “Latino*”[All Fields] OR “Latina*”[All Fields] OR “Spanish speaking”[All Fields] OR “Healthcare Disparities”[Mesh] OR “Healthcare Disparities”[All Fields])
WEB OF SCIENCE	(Multiple Myeloma OR Plasmacytoma) AND (Hispanic Americans OR Hispanic OR Hispanics OR Spanish Americans OR Latino* OR Latina* OR Spanish speaking OR Healthcare Disparities)

### Study Abstraction and Analysis

Two authors (A.A.G., S.A.H) with clinical backgrounds in clinical research, medical oncology, hematology, and internal medicine participated in study selection. Research studies were accepted if they included Hispanic Americans as part of the studied population. Review articles, editorials, studies that solely described protocols, non-research letters, abstracts of congresses, and books were excluded. Also excluded were health disparities studies which did not report specific results on Hispanic Americans and/or included their results under ‘others’.

The studies’ abstracts were reviewed for eligibility, and full papers were reviewed if eligibility was not clear from the abstract review alone. Each study was categorized under a specific domain that included the major study aim and/or finding. Categories included incidence, socioeconomic status, treatment, clinical trial participation and mortality.

## Results

**Fig. 1:**
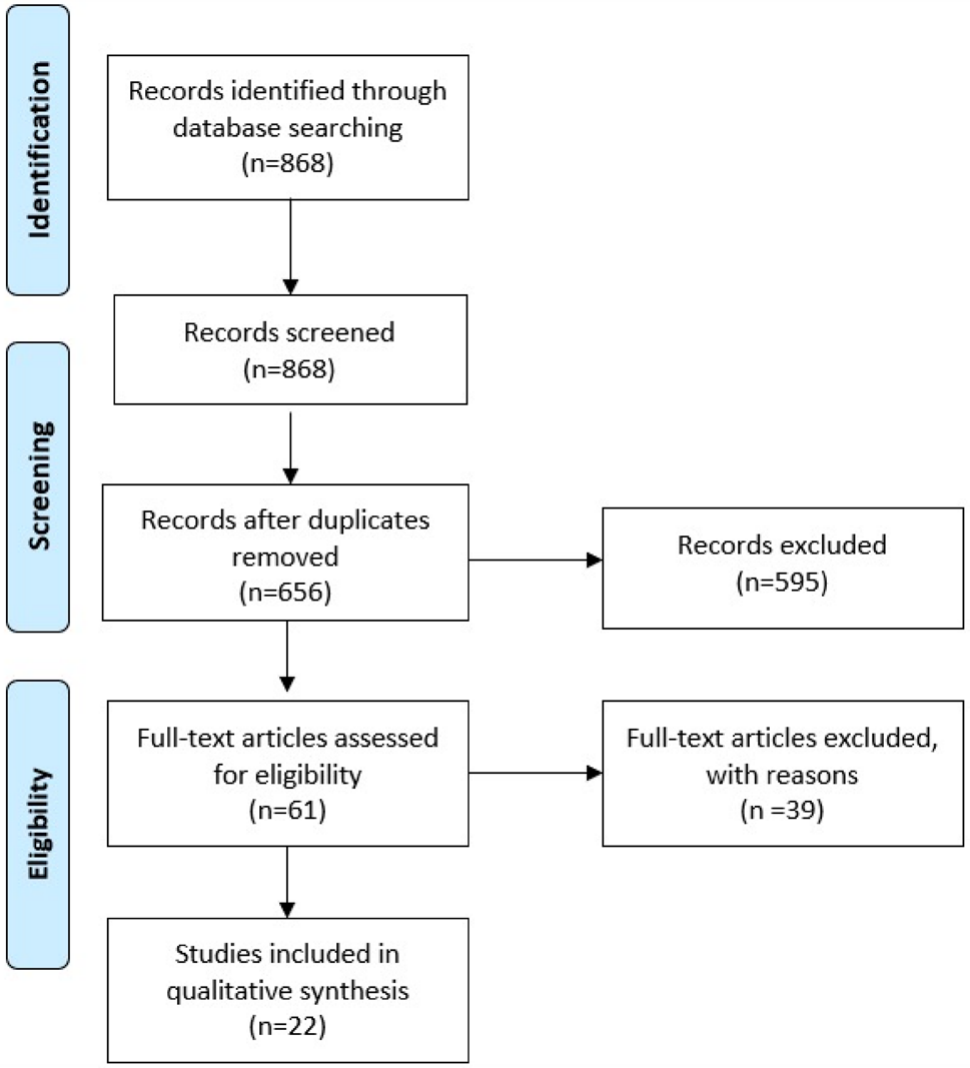
Flow diagram of database search

Our wider broad search retrieved a total of 868 records, of which 212 reports were related to the same patients. After in-depth screening, 595 reports were excluded for not fulfilling the inclusion criteria (Fig. 1). Of the remaining 61 records, 39 reports were also excluded after full text review for not meeting the eligibility criteria. A total of 22 studies focused on Hispanic Americans with MM were included [5, 7–26]. We categorized the articles based on topic, year of publication and author. Table 2 summarizes the characteristics and conclusions of the articles according to the domain.

**Table 2:** Articles included in the review

Author	Year	Results	Title
*Incidence*			
Kaur et al. [7]	2021	Hispanics had a higher incidence of MM compared to NHW. The median age at presentation was 5 years younger in Hispanics than NHWs, and patients were more likely to present with renal dysfunction. Hispanics had a higher proportion of Revised International Staging System (R-ISS) stage I disease than NHW and NHB. At the same time, there was no difference in cytogenetics between Hispanics and NHB/NHW. In the multivariate analysis, only high-risk disease, and response to first-line therapy significantly affected survival	Multiple myeloma in Hispanics: Incidence, characteristics, survival, results of discovery, and validation using real-world and connect MM registry data
Castañeda-Avila et al. [8]	2020	The median age at diagnosis was the same among non-Hispanics in the SEER population and Puerto Rico at approximately 69 years old, but slightly lower among Hispanics in SEER at 65 years (*p*-value < 0.01)	Trends in cause of death among patients with multiple myeloma in Puerto Rico and the United States SEER population, 1987–2013
		The mean age at death from both MM and other causes was higher among non-Hispanics in SEER when compared to both Puerto Ricans and Hispanics in SEER (*p*-value < 0.001). Furthermore, Hispanics in SEER had a younger median age at death from both MM and other causes than Puerto Ricans	
Costa et al. [9]	2017	MM incidence did not increase significantly among NHB women and Hispanics. Improvement in 5-year RSRs (1993–1997 vs 2008–2012) was seen among patients of all age and race/ethnicity groups	Recent trends in multiple myeloma incidence and survival by age, race, and ethnicity in the United States
		Among patients 65–74 years of age, 10-year RSRs improved for NHWs (11.3% vs 20.5%) and Hispanics (10.6% vs 20.2%), but not for NHBs (12.6% vs 19.5%)	
Ailawadhi et al. [10]	2012	Hispanics had the youngest median age at diagnosis (65 years), and Whites had the oldest (71 years) (*p* < 0.001). Hispanics had the worst median OS (2·4 years). These trends were more pronounced in patients older than 75 years	Outcome disparities in multiple myeloma: A SEER-based comparative analysis of ethnic subgroups
*Socioeconomic status*			
Castañeda-Avila et al. [11]	2021	Lower proportions of cases diagnosed with MM who lived in a neighborhood with high SES were Hispanics or Blacks (7% and 8%) cases than the proportion of cases from low SES neighborhoods (17.5% and 32%)	Differences in survival among multiple myeloma patients in the United States SEER population by neighborhood socioeconomic status and race/ethnicity
		Among Hispanic adults diagnosed with MM, there was not a significant difference in cancer-specific survival between SES categories	
Evans et al. [12]	2021	The percentage of patients residing in ZIP Code areas with low-income levels were different across race and ethnic groups (45% among Asians, 64% among Caucasians, 78% among all Hispanics, and 84% among Black patients; *p* < 0.001). Similarly, the percentage of patients residing in ZIP Code areas with low education levels was different across race and ethnic groups (72% among Asians, 70% among Caucasians, 92% among all Hispanics, and 90% among Black patients; *p* < 0.001)	The impact of socioeconomic risk factors on the survival outcomes of patients with newly diagnosed multiple myeloma: a cross-analysis of a population-based registry and a tertiary care center
Kamath et al. [13]	2020	Strong neighborhood-level correlations exist between incidence and mortality rates and the high prevalence of residents of Latin American birth. Higher MM mortality also correlated with Hispanic ethnicity, obesity, diabetes, poverty, HIV/AIDS, air benzene concentration, and indoor pesticide use	Where you live can impact your cancer risk: a look at multiple myeloma in New York City
*Treatment*			
Joshi et al. [14]	2021	Underuse of maintenance therapy was highest among patients with NHB and Hispanic backgrounds (25.2% and 23.7%, respectively vs. 12.5% and 16.4% among Asian and NHW patients, respectively)	Multiple myeloma, race, insurance and treatment
		Multivariate modeling of unplanned interruptions in treatment found that patients with Medicaid and those who were Hispanic, or Black were most affected	
Zhou et al. [15]	2021	White patients had higher rates of intravenous bisphosphonate initiation (56%), compared with NHB (45%), Hispanic (50%), and API (47%),	Ethnic disparities in Intravenous bisphosphonate use among older patients with multiple myeloma enrolled in Medicare
		Black patients had a lower duration of IV bisphosphonate treatment compared with other ethnic groups (median 267 days v 294 [White], 293.5 [API], and 282.5 [Hispanic and Latino])	
		Black patients had a slightly lower mean number of cumulative IV bisphosphonate doses (14.0), compared with White (15.9 doses), API (15.6 doses), and Hispanic and Latino patients (14.7 doses)	
		The SRE rates were highest among Hispanic patients (49.5%), followed by White (49.1%) and API (45.1%), whereas Black patients had the lowest rates of SREs (38.2%). In multivariable competing risk regression models, non-White patients had significantly delayed or no initiation of IV bisphosphonates compared with White patients	
Ailawadhi et al. [16]	2019	Hispanics had a longer time from MM diagnosis to novel therapy initiation vs whites (median: 4.6 vs 2.7 months, respectively). ASCT rate within 1 year of MM diagnosis rose among whites and African Americans (*p* < 0.05), but not Hispanics, who were less likely to receive ASCT vs whites	Ethnic disparities in treatment patterns and outcomes among patients with multiple myeloma: A SEER-Medicare analysis
Ailawadhi et al. [42]	2018	There was higher receipt of immunomodulatory drugs among Hispanics and Asians (*p* < 0.001). Medicare claims were highest at any time after MM diagnosis for Hispanics. Over time, Medicare claims increased most steadily for Hispanics (*p* < 0.001)	Trends in multiple myeloma presentation, management, cost of care, and outcomes in the Medicare population: A comprehensive look at ethnic disparities
Schriber et al. [18]	2017	The stem cell transplant utilization rate increased across all groups from 2008 to 2014. The increase was substantially lower among Hispanics (8.6–16.9%) and NHBs (12.2–20.5%) than for NHWs (22.6–37.8%). Fewer patients over 60 were transplanted in Hispanic (39%) and non-Hispanic Blacks (42%) vs. non-Hispanic Whites (56%)	Hispanics have the lowest stem cell transplant utilization rate for autologous hematopoietic cell transplantation for multiple myeloma in the United States: A CIBMTR report
		More Hispanic (57%) vs. NHBs (54%) and NHWs (52%) (*p* < 0.001) had stage III disease	
		More Hispanics (48%) vs. NHBs (45%) and NHWs (44%) were in ≥ very good partial response pre-transplant (*p* = 0.005)	
		Race and ethnicity did not impact post-allogeneic hematopoietic cell transplantation (AHCT) outcomes	
Ailawadhi et al. [19]	2017	There was significantly higher thalidomide use among Hispanics. Hispanics had the highest median number of days to the first dose of bortezomib and the lowest utilization of SCT. Hispanics were the only groups without notable increases in lenalidomide	Ethnic disparity in utilization of therapeutic modalities among multiple myeloma patients: a SEER-Medicare analysis
Costa et al. [20]	2015	Age-adjusted RUR was 1.17 among NHW, higher than in Hispanics [0.64 (0.60–0.69), *p* < 0.002]. There was higher AHCT utilization in men than in women among Hispanics but not among NHW, NHB or Asians. Sex disparity prevents 1.3% of potential AHCTs in MM (10.4% among Hispanics). Ethnic disparities prevent 13.8% of AHCTs (44.7% in Hispanic and Asians, 39.9% in NHBs)	Disparities in utilization of autologous hematopoietic cell transplantation for treatment of multiple myeloma
Al-Hamadani et al. [21]	2014	In multivariable analysis, patients with Hispanic ethnicity (0.78) were the least likely to receive AHCT	Use of autologous hematopoietic cell transplantation as initial therapy in multiple myeloma and the impact of socio-geo-demographic factors in the era of novel agents
*Clinical trial*			
Jayakrishnan et al. [22]	2021	Hispanic patients were 5.6% of the total of enrolled patients to systemic therapy and survival for patients with multiple myeloma	Disparities in the enrollment to systemic therapy and survival for patients with multiple myeloma
Duma et al. [23]	2018	NHWs were more likely to be enrolled in clinical trials (EF 0.18%) than NHBs (EF 0.06%, *p* < 0.0001) and Hispanic patients (EF 0.04%, *p* < 0.0001)	Representation of Minorities and Elderly Patients in Multiple Myeloma Clinical Trials
Ailawadhi et al. [17]	2018	Hispanics had the smallest proportion of patients on trials utilizing novel therapeutic agents. While adverse demographic (increased age) and clinical (performance status, stage, anemia, kidney dysfunction) factors were associated with inferior survival, patient race-ethnicity did not have an effect on objective response rates, progression-free, or overall survival	Disease and outcome disparities in multiple myeloma: Exploring the role of race/ethnicity in the Cooperative Group clinical trials
*Mortality*			
Al Hadidi et al. [5]	2021	MM-related in-hospital mortality was significantly higher in Hispanics compared to NHWs and NHBs. Using average annual percent change, there is a statistically significant decline of in-hospital mortality among all MM patients except NHBs, who had the highest inpatient mortality in recent years	Health disparities experienced by Black and Hispanic Americans with multiple myeloma in the United States: a population-based study
Ailawadhi et al. [24]	2019	Five-year and 10-year RSR improved for patients aged > 40 years diagnosed in the 3 time periods for NHW, NHB, and Hispanics (all *p* < 0.0001), as well as both genders (*p* < 0.0001) evaluated separately	Survival trends in young patients with multiple myeloma: a focus on ethnic minorities
		For an evaluation by race/ethnicity, the 5- and 10-year RSRs improved significantly over time for NHW and NHB (all *p* < 0.0001), but not for Hispanics. Although the RSRs did improve for Hispanics over time, the improvement was not statistically significant	
Costa et al. [25]	2016	Hispanic and NHB individuals were found to have a higher risk of death compared with NHW individuals, despite their earlier age at the time of diagnosis. However, after adjusting for marital status, insurance status, county-level household income, sex, and age, race/ethnicity was found to no longer significantly influence survival, suggesting that the apparent impact of race/ethnicity on O.S. may be due to the disparate distribution of sociodemographic factors observed among the different races/ethnicities rather than the race/ethnicity construct itself	Impact of marital status, insurance status, income, and race/ethnicity on the survival of younger patients diagnosed with multiple myeloma in the United States
Pulte et al. [26]	2014	The greatest improvements were observed for patients aged 15–49, for whom survival increased by 16.8% units for NHW and 14.4% units for A.A., whereas improvement was less pronounced and not statistically significant in Hispanics and API. Excess mortality hazard ratios was 1.25 (95% CI: 1.11–1.41) for Hispanics compared to NHW in 2006–2009. Although survival increased greatly for NHW with myeloma between 1998–2001 and 2006–2009, smaller increases were observed for people of other ethnic groups. Persistent excess mortality was seen for African American and Hispanic patients with MM	Recent improvement in survival of patients with multiple myeloma: variation by ethnicity

*NH* non-Hispanic; *NHW* non-Hispanic white; *NHB* non-Hispanic black; *API* Asian Pacific Islander; *ASCT* autologous stem cell transplant; *RSR* relative survival rate; *SES* socioeconomic status

The number of publications varied over time with the highest number of studies (32%) published in 2021. The articles were published in 16 different journals with 179 different contributing authors. Fifty-nine percent of the included articles reported worse outcomes for Hispanic Americans when compared with other ethnic groups.

The incidence of MM in Hispanics is higher and with a median age at presentation 5 years younger than in non-Hispanic Whites [7, 10]. Some prior studies suggested no difference in incidence of MM in Hispanics when compared to non-Hispanic Whites [9]. There was no difference in adverse risk cytogenetics between Hispanics and non-Hispanic Whites and non-Hispanic Blacks [7]. Socioeconomic status (SES) reflected by residence zip code and poverty level was shown to be different among different ethnic groups with higher proportion of Hispanic Americans living in lower SES zip codes and zip codes with low education levels [11–13]. Hispanic Americans were found to receive less MM maintenance therapy and less supportive therapies such as bisphosphonates [14, 15].

A longer time from MM diagnosis to novel therapy initiation was more prevalent in Hispanic Americans when compared to non-Hispanic Whites [16]. Autologous stem cell transplantation (ASCT) use in Hispanic Americans is lower than non-Hispanic Whites and, despite the incremental use of ASCT from 2008 to 2014, Hispanic Americans had the lowest rates of ASCT when compared to all other ethnic groups [16, 18, 19, 21].

Enrollment in MM clinical trials was lower in Hispanic Americans [17, 22, 23]. The rate of in-hospital mortality was higher in Hispanic Americans when compared to other ethnic groups [5]. Despite the earlier age of diagnosis, Hispanic Americans were found to be at higher risk of death, which may be related to lower SES [25]. Improvement in MM survival, in this treatment landscape era of improved therapy options, was least pronounced in Hispanic Americans [26].

## Discussion

We found that Hispanic Americans had a higher incidence of MM compared to non-Hispanic whites. Our review found that Hispanic Americans with MM tend to live in areas with low SES and education levels. This is in line with prior data suggesting that Hispanic Americans are more likely to live in poverty than non-Hispanic Whites. Generally, lower SES among people of color in the U.S. stems from long-term structural racism or racism that is reinforced by discriminatory laws, economic policies, and societal and cultural norms [27].

Many Hispanic patients face financial, structural, and personal barriers to health care. In fact, Hispanic people continue to be the least likely to have health insurance of any major ethnic group [27–29]. When they have equal access to therapy, Hispanic Americans have survival similar to non-Hispanic Whites and African Americans [7]. Hispanic patients might not experience similar benefits from the introduction of novel therapies and even standard treatment, as their outcomes are worse than non-Hispanic Whites and, in some cases, worse than the rest of the other ethnic groups, at least in part because they received novel therapies later than non-Hispanic Whites [16].

Gender-dependent differences may influence the primary genetic events of MM. Women have a poor prognosis with higher prevalence of immunoglobulin heavy chain gene translocations, which was found to be associated with inferior overall survival [30]. However, no obvious differences in significant measures of genomic variation were found in Hispanic Americans when compared to non-Hispanic Whites [31].

Hispanic patients continue to be the smallest proportion of patients on trials utilizing novel therapeutic agents in MM [22]. Cancer is the leading cause of death among Hispanics. However, only 1.3% of eligible Hispanic cancer patients participate in cancer-related clinical trials [32, 33]. Patient education on clinical trials and materials in Spanish can improve their enrollment [34].

Hispanics are the United States' largest and fastest-growing minority group. They currently make up about 15 percent of the U.S. population [4]. However, only 5.8% of active physicians are Hispanic [35]. Moreover, Hispanic doctors made only 3.3% of awardees from the seven major Hematology-Oncology societies [36–38]. As a possible solution, it would be of public interest to diversify the medical workforce. Minority patients are more likely to choose a minority physician and are more satisfied with their care when it is provided by a minority physician [39, 40].

Our review shows that the highest number of studies pertaining to disparities in the MM Hispanic American population were published in 2021 (32%). This likely reflects an increased awareness regarding the scope of this problem and a call to action to combat these prevalent disparities. Studies from Latin America suggested a poor progression-free survival (PFS) among patients with relapsed MM and a slower uptake of newer therapies in public clinics [41].

Our study has some limitations. While we conducted a rigorous, scoping review of existing literature, we did not assess the quality of the included studies. We may have missed published literature that was not captured using our search terms and some publications may have been misclassified. Additionally, the included studies may have varied in their definition of “Hispanics”. For example, some specifically looked at Hispanic Whites and Hispanic Blacks. Nevertheless, our study summarizes the current scope of health disparities experienced by Hispanic Americans and highlights areas that require immediate attention.

## Conclusion

We found a growing body of articles that studied health disparities that affect Hispanic Americans with MM. Most of the articles reported worse clinical outcomes for Hispanic Americans compared to other ethnic groups. There is an urgent need to implement systemic and structural solutions to current barriers precluding equitable access to care for Hispanic patients.

## Data Availability

The data supporting this study's findings are available on request from the corresponding author.

## Abbreviations

MM: Multiple myeloma
PRISMA: Preferred Reporting Items for Systematic Reviews and Meta-Analyses
MESH: Medical Subject Headings
SES: Socioeconomic status
ASCT: Autologous stem cell transplantation
NH: Non-Hispanic
NHW: Non-Hispanic white
NHB: Non-Hispanic black
API: Asian Pacific Islander
RSR: Relative survival rate
